# Centrally acting antihypertensives and alpha-blockers in people at risk of falls: therapeutic dilemmas—a clinical review

**DOI:** 10.1007/s41999-023-00813-x

**Published:** 2023-07-12

**Authors:** T. J. Welsh, A. Mitchell

**Affiliations:** 1 University of Bristol, Bristol, UK; 2 RICE—The Research Institute for the Care of Older People, The RICE Centre, Royal United Hospital, Bath, UK; 3 Royal United Hospitals Bath NHS Foundation Trust, Bath, UK; 4 Pharmacy Department, University Hospitals Plymouth NHS Trust, Plymouth, UK; 5 Department of Life Sciences, University of Bath, Bath, UK

**Keywords:** Falls, Deprescribing, Geriatric, Hypertension

## Abstract

**Purpose:**

The aim of this clinical review was to summarise the existing knowledge on the adverse effects of alpha-blockers and centrally acting antihypertensives, the effect these may have on falls risk, and guide deprescribing of these medications.

**Methods:**

Literature searches were conducted using PubMed and Embase. Additional articles were identified by searching reference lists and reference to personal libraries. We discuss the place of alpha-blockers and centrally acting antihypertensives in the treatment of hypertension and methods for deprescribing.

**Results:**

Alpha-blockers and centrally acting antihypertensives are no longer recommended for the treatment of hypertension unless all other agents are contraindicated or not tolerated. These medications carry a significant falls risk and non-falls risk-associated side effects. Tools to aid and guide de-prescribing and monitoring of the withdrawal of these medication classes are available to assist the clinician including information on reducing the risk of withdrawal syndromes.

**Conclusions:**

Centrally acting antihypertensives and alpha-blockers increase the risk of falls through a variety of mechanisms—principally by increasing the risk of hypotension, orthostatic hypotension, arrhythmias and sedation. These agents should be prioritised for de-prescribing in older frailer individuals. We identify a number of tools and a withdrawal protocol to aid the clinician in identifying and de-prescribing these medications.

## Key summary points

**Purpose:**

To summarise the evidence for deprescribing alpha-blockers and centrally acting antihypertensives in older people at risk of falls and to assist clinicians in deciding how to safely de-prescribe these agents.

**Findings:**

Alpha-blockers and centrally acting antihypertensives are no longer recommended for the treatment of hypertension unless all other agents are contraindicated or not tolerated. Multiple tools identify these agents as potentially inappropriate and recommend deprescribing. A number of protocols have been developed to safely guide deprescribing.

**Message:**

Safer antihypertensives are available that are associated with less adverse effects. Decision aids such as STOPPFalls can assist de-prescribing.

## Introduction

Hypertension is the most important cardiovascular risk factor with the greatest impact on mortality [1–3]. Guidelines across the globe stress the importance of its detection and treatment. High blood pressure is very common. Prevalence increases with age [4] and approximately 80% of those aged over 80 are hypertensive [5]. Multiple large-scale randomised controlled trials (RCTs), such as the Hypertension in the Very Elderly Trial (HYVET), have demonstrated health benefits from medications that lower blood pressure [4, 6], and increasingly guidelines are advocating lower and lower target blood pressures even amongst the oldest old [7–9]. However, there has been concern amongst clinicians, and within the academic literature, that the benefits of antihypertensives may be attenuated in frailer older people whilst the risks, such as falls, may be higher—thus creating a prescribing dilemma [10]. Evidence associating falls with antihypertensives is variable, reflecting the multitude of different antihypertensives and variation in observational studies. Observational data highlight the period of medication initiation or dose increase as being the period of greatest risk [11]. Alpha-blockers and centrally acting antihypertensives are frequently highlighted as particularly problematic classes in cognitively and physically frailer people.

However, alpha-blockers and centrally acting antihypertensives still form part of the antihypertensive pharmacopoeia albeit less prominently than in the past [12]. Their use is now restricted to that of an add-on therapy in cases of resistant hypertension. Data on the extent of current usage are sparse. However, a large retrospective cohort study in Canada identified 2% of women aged ≥ 65 years with a diagnosis of hypertension were prescribed an alpha-blocker [13]. Alpha-blockers for the management of prostatism are frequently prescribed and clinicians are more likely to encounter them in this context. This review will, however, focus on alpha-blockers whose primary indication is hypertension. Centrally acting antihypertensives were widely used in the earliest decades of antihypertensive treatment when other treatments were not available, but are less frequently used now, principally because of their poorer tolerability relative to the newer major classes of drugs [14]. The National Institute for Health and Care Excellence (NICE) no longer mentions moxonidine or clonidine, and European and International guidelines list moxonidine as an alternative 4th-line antihypertensive only if spironolactone is not an option and only under specialist use [7, 15]. Methyldopa for hypertension is advised for use only in pregnancy and so is unlikely to be encountered for this indication in clinical practice by the average geriatrician. However, clinicians will of course encounter patients prescribed methyldopa in the context of Parkinson’s disease—a context which lies outside the scope of this discussion.

This clinical review was informed by a literature search conducted in PubMed and Embase initially in May 2022 and updated in November 2022 including primary research, systematic reviews and meta-analyses. Summary Product Characteristics (SmPC), relevant hypertension guidelines, personal reference libraries and the British National Formulary (BNF) were also utilised. Keywords for the searches included “falls”, “doxazosin”, “prazosin”, “terazosin”, “indoramin”, “moxonidine”, “clonidine”, “methyldopa”, “deprescribing”, “older adults” and appropriate variations of these terms.

This review will explore the fall-related risks of alpha-blockers and centrally acting antihypertensives used to treat hypertension. We provide an evidence review which may be of benefit to the clinician caught in a dilemma with regard to antihypertensive prescription. We review the factors that should be explored if de-prescribing is being considered and outline a pointers for de-prescribing.

## Medication review and reconciliation

Establishing the indication for the medication is important—is the drug in question being used primarily for high blood pressure or for another condition? Alpha-blockers and centrally acting antihypertensives are now not generally recommended for the management of hypertension. They sit as fourth-line agents in most guidelines. For alpha-blockers, this is largely due to The ALLHAT trial which evaluated the alpha-blocker doxazosin as first-line antihypertensive therapy against diuretic treatment [16]. The trial had to be stopped early due to the increased risk of adverse cardiovascular events with doxazosin compared with chlorthalidone [16]. However, some older patients may still be prescribed over these agents as a legacy of historic prescribing trends or due to intolerance of alternative antihypertensives. For the practicing clinician working with older people, these medications will more frequently be used for alternative diagnoses. Thus, alpha-blockers will be most frequently encountered as a treatment for benign prostatic hyperplasia (BPH), and methyldopa will be encountered as part of the treatment regimen for Parkinson’s disease and related conditions.

## Measuring blood pressure and targets

Blood pressure must be assessed carefully. White coat hypertension affects around one in four older people [17], and home blood pressure measurement or ambulatory measurements are preferred to those carried out in clinic as they are more reliable [18] and more accurate [19]. Postural blood pressure measurements must be carried out. In those with orthostatic hypotension, standing blood pressure measurements should be used to guide therapy decisions [20]. The European and UK guidelines suggest treating blood pressure over 150/80 mmHg in those aged over 80 [7, 20] and in frailer groups, younger than 80 years (European guideline only) [7]. Guideline target blood pressure varies depending on co-morbidities and guideline. NICE recommends a target clinic BP of < 150/80 mmHg [20], the ESC/ESH a systolic of 130–139 mmHg if tolerated [7], and the International Society of Hypertension guideline < 140/90 mmHg for the people aged over 65 with the proviso to consider an individualised target in frailty and likely tolerability [15].

The guidelines all try to provide an evidence-based rationale for the suggested regimes with medication choice and blood pressure target adjusted to suit the comorbidities, e.g., heart failure. However, the typical trial participant does not represent the frail, multi-morbid older individual who the practicing geriatrician will most frequently encounter [21]. It is also important to bear in mind that in every randomised trial of antihypertensive medication, greater blood pressure control has led to a reduction in adverse vascular outcomes such as stroke or MI. The SPRINT trial demonstrated that greater control led to even greater improvements [22]. In the robust older patient, there seems little reason to deviate from guideline advice; however, in the frailer older adult—perhaps presenting with falls—the situation is different and attenuated target blood pressures or no target at all—with a focus on outcomes meaningful for the patient becomes more important.

## Cautions and contraindications

As part of any medication review, it is important to consider any potential contraindications, and antihypertensive medications are no exception. For alpha-blockers, orthostatic hypotension and established heart failure (particularly if related to aortic stenosis) are contraindications [23]. The presence of these conditions should therefore prompt consideration of de-prescribing. For the centrally acting antihypertensives, heart block and sick sinus syndrome are contraindications for both moxonidine and clonidine [23]. Moxonidine is also contraindicated in bradycardia and severe heart failure [23]. For methyldopa, depression should act as a contraindication as well as rarer problems, such as porphyria, paraganglioma and pheochromocytoma [23]. All the centrally acting antihypertensives and alpha-blockers are flagged by the ‘screening tool of older people's prescriptions (STOPP)’ criteria as potentially inappropriate medications—particularly in the context of postural hypotension—and should be reviewed accordingly [24].

Drug–drug interactions are common for methyldopa with a higher risk of hypotension reported when used in conjunction with antihypertensives and other medications with hypotensive side effects (e.g., antipsychotics) [23]. Moxonidine and clonidine have similar issues but also have the potential to increase the risk of drowsiness, confusion and CNS depression if used in conjunction with other neuro-active medications [23]. The alpha-blockers have multiple drug–drug interactions with increased issues with hypotension reported with multiple drug classes [23].

## Falls and fall-related adverse events

The association between antihypertensives as a group and fall risk is mixed. Multiple systematic reviews have failed to provide a definitive answer. Stable long-term use of antihypertensives in otherwise well older people does not appear to be associated with falls [25]. However, the risk of falling does increase for the 24-h period following antihypertensive initiation or dose increase, and this persists for 21 days in the case of diuretic antihypertensives [25].

Studies that have evaluated the association between alpha-blockers and falls/fractures report variable findings. Hiremath and colleagues found no association with falls due to alpha-blockers in older women, hazard ratio (HR) 1.02 (95% CI 0.92–1.13) compared to other antihypertensives [13]. Whilst Welk and colleagues found a significant association between new prostate-specific alpha-blocker prescription for men and fracture odds ratio (OR) 1.16 (95% CI 1.07–1.21) and falls OR 1.14 (95% CI 1.07–1.21) [26]. Meta-analyses by Ang and colleagues identified no increased falls risk with alpha-blockers (OR 0.92 (95% CI 0.76–1.12) [27], whilst de Vries and Mansbart and colleagues found a trend towards increased risk (OR 1.62 (0.76–3.45) and OR 1.08 (95% CI 0.99–1.17) respectively) [28, 29].

We identified no recent systematic reviews of falls and centrally acting antihypertensives. The most recent study identified was from 1999 (including papers published 1966–1996)—Leipzig and colleagues identified a trend towards an association between centrally acting antihypertensives and falls OR 1.16 (95% CI 0.87–1.55) [30]. The lack of more recent reviews on this drug class reflects the move to alternative classes of antihypertensives with better outcome data and fewer reported side effects. Where centrally acting antihypertensives are discussed in the context of blood pressure in older people, it is to highlight the risks of orthostatic hypotension and significant side effects [31]. Both STOPP and Beer’s criteria highlight them as potentially inappropriate [24, 32].

Both centrally acting antihypertensives and alpha-blockers are associated with a number of adverse effects known to be risk factors for falling. These can be grouped into cardiac, sleep-related and neuro-psychiatric. Table 1 details the frequency of these events.

**Table 1:** Incidence of adverse effects with alpha-blockers and centrally acting antihypertensives that could increase the risk of falls. Source: cognitive enhancer SmPCs [58]

Anti-hypertensive	Dizziness/vertigo	Hypotension	Syncope	Drowsiness	Blurred vision	Sleep disorders	Arrhythmia
**Alpha-blockers**							
Doxazosin	+ + +	+ + +	+ +	+ + +	+	+ +	+ + +
Prazosin	+ + +	+ + +	+ + +	+ + +	+ + +	+ +	+ +
Terazosin	+ + +	+ + +	+ +	+ + +	+ + +	+ + +	+ +
Indoramin	Frequency not known	Frequency not known	Frequency not known	Frequency not known	No data	Frequency not known	No data
**Centrally acting antihypertensives**							
Moxonidine	+ + +	+ +	+ +	+ + +	No data	+ + +	+ +
Clonidine	+ + + +	+ + + +	No data	+ + + +	Frequency not known	+ + +	+ +
Methyldopa	Frequency not known	Frequency not known	No data	Frequency not known	No data	Frequency not known	Frequency not known

^+^ < 1/1000

^++^1/100–1/1000

^+++^1/10–1/100

^+ + + +^ > 1/10

## Cardiovascular side effects

Hiremath and colleagues found an association between alpha-blockers and hypotension HR 1.71 (95% CI 1.33–2.2) and hypotension-related events HR 1.1 (95% CI 1.01–1.2) [13]. Chrischilles and colleagues reported that up to 50% of people given either terazosin, doxazosin or prazosin for BPH had to discontinue the alpha-blocker [33]. This was more common in people who were taking other antihypertensive medications. Hypotension-related adverse events were more common in those on alpha-blockers than in non-users [33].

The main cardiovascular side effects of the centrally acting antihypertensives are hypotension and orthostatic hypotension, but both clonidine and moxonidine can cause bradyarrhythmias [34, 35].

## Neuro-psychiatric symptoms and sleep disorders

A number of neuro-psychiatric side effects are listed by the manufacturers as occurring at a frequency of 0.1–10% in people treated with both alpha-blockers and centrally acting antihypertensives [34–39]. For alpha-blockers, fatigue is the most common affecting between 5 and 15% of patients [40]. Sleep disturbance and anxiety can occur with alpha-blockers, but are reportedly mild [40]. Both fatigue and sleep disturbance can contribute to falls risk.

Neuro-psychiatric adverse events are more common with centrally acting antihypertensives. Sedation is common with both clonidine and methyldopa, occurring in over a third of patients [34, 39–41]. Depression is common with clonidine, and hallucinations and confusion have also been reported although less frequently [34, 41]. Methyldopa is associated with insomnia (incidence 0.8%), nightmares (incidence 2%), mild psychoses and depression (incidence 3.6%) [39, 42].

## Evidence on de-prescribing alpha-blockers and centrally acting antihypertensives

We have been unable to identify any de-prescribing trials in older people that focus specifically on alpha-blockers or centrally acting antihypertensives. This situation is balanced, to a degree, by the absence of RCT evidence for continuing these medications in frailer older people. Indeed, the evidence for alpha-blockers is questionable even in non-frail groups and lacking for centrally acting antihypertensives.

When considering antihypertensives more generally, the balance of risks and benefits in physically or cognitive frail older people has been questioned in the research literature for some time [43]. Indeed, there is observational evidence that the benefits of blood pressure lowering may be attenuated and the risks increased in these groups. For instance, the PARTAGE group found an association between increased mortality and a systolic BP below 130 mmHg in care home residents taking two or more antihypertensives [44]. The Leiden 85 + study found an association between cognitive decline and low blood pressure in patients taking antihypertensives [45], a finding replicated by Mossello and colleagues [46].

Regardless of frailty or age, individuals with controlled blood pressure may no longer require treatment. Van de Wardt and colleagues review on antihypertensive withdrawal found that 40% (95% CI 38–42%) of people remained below treatment threshold after antihypertensive medication was withdrawn at 1 year [47]. Monotherapy and lower blood pressure predicted success [47].

Crisafulli and colleagues conducted a systematic review of antihypertensive de-prescribing trials [48]. They identified two open label de-prescribing RCTs—one conducted in England (OPTIMISE) [49] and the other in the Netherlands (ECSTATIC) [50], and neither focussed on frail older people. OPTIMISE found that a reduction in antihypertensive medication in community dwellers aged over 80 was not associated with a significant change in blood pressure or adverse events during 12 weeks of follow-up [49]. ECSTATIC found no significant difference in cardiovascular risk over 2 years of follow-up in people aged between 40 and 70 (with a low baseline cardiovascular risk) assigned to either usual care or medication reduction [50]. These studies demonstrated that even in non-frail groups, antihypertensives can be de-prescribed effectively.

## Decision aids for de-prescribing

Multiple tools exist to help the clinician identify potentially inappropriate medications [51]. Such tools include the American Geriatric Society Beer’s criteria [32], STOPP/START [24], STOPPFall [52] and FORTA [53]. All these tools highlight centrally acting antihypertensives as potentially inappropriate [24, 32, 52, 53]. STOPP/START and FORTA go further in stating that they should be avoided in frailer older people with FORTA classifying them as ‘D’—“Do not” [24, 53]. Alpha-blockers for hypertension are also identified as being potentially inappropriate by all these tools. FORTA classifying them as ‘C’—“Careful” [53] and STOPPFall highlighting them as a medication class of concern in those at risk of falls [52]. These tools are helpful in assisting the clinician to identify potentially inappropriate medications but have little to say about how the medication should be stopped—except STOPPFalls which provides some guidance [52].

We identified four tools to guide the clinician in the practicalities of prescribing/de-prescribing antihypertensives in older frailer people. Two developed in Australia and two in Europe.

Benetos and colleagues advocate stratifying antihypertensive treatment according to frailty and functional status [54]. Their objective was to avoid exclusion based solely on advanced age and to avoid aggressive therapy targeting only life prolongation. They identify three groups: (i) Older adults with preserved functional status (Preserved Function Profile)—consider full therapy, (ii) Older adults with moderate functional decline and preserved autonomy for activities of daily living (ADL) (Loss Of Function/Preserved ADL Profile)—should undergo detailed frailty/functional assessment and tailor antihypertensive therapy accordingly, (iii) Older Adults With Significant Loss of Function and Loss of Autonomy for ADL and Limited Life Expectancy (Loss of Function and Altered ADL)—The frailest group and the most vulnerable to adverse side effects from treatment [54]. Treatment in this group should be reassessed with symptom relief and quality of life the primary goal. De-prescribing should be actively considered.

Primary Health Tasmania produced a guide to de-prescribing antihypertensive agents [55]. The guidance recommends considering dose reduction or cessation of antihypertensives in the frail older and/or immobile patient, in those at high risk of falls, and in those with postural hypotension [55]. De-prescribing should be balanced against factors which may favour continuing a medication such as multiple cardiovascular risk factors or recent stroke. The guideline highlights specific issues with alpha-blockers—hypotension, peripheral oedema, worsening of stress incontinence in women; centrally acting agents—sedation, constipation, dry mouth which may prompt a discussion around de-prescribing [55]. The guideline also highlights concerns around possible withdrawal syndromes when medication is discontinued. This could include peripheral oedema, tachycardia, rebound hypertension, worsening heart failure or ischaemic heart disease. On this basis, the guidance recommends dose tapering by 25% every month [55].

Scott and colleagues presented a decision framework that coalesces around five steps [31]: (i) decide on the therapeutic goals with shared decision-making, (ii) Estimate cardiovascular risk and or life expectancy, (iii) Accurately measure the blood pressure and orthostatic pressures, (iv) identify threshold and target blood pressures, (v) consider situations for de-prescribing, e.g. over 80 no cardiovascular disease, severe frailty, functional limitations, cognitive impairment, history of syncope or falls and life expectancy less than 1 year [31].

When a decision has been made to de-prescribe, then a logical, evidence-based approach to medication withdrawal is needed. Harrison and colleagues conducted a review of the literature, guidelines and British National Formulary (BNF) to develop an antihypertensive withdrawal procedure and monitoring procedure [56]. This formed part of a broader study examining the feasibility of withdrawing antihypertensive medication in people with dementia and well-controlled hypertension [57]. However, it does have potentially wider applications. This protocol provides an evidence-based tool for clinicians to use as an aid to the practical aspects of dose reduction, treatment withdrawal and monitoring [56].

## Conclusions

Hypertension is one of the commonest long-term health conditions. Multiple studies have shown reductions in morbidity and mortality with appropriate antihypertensive treatment. Alpha-blockers and, to a lesser extent, centrally acting antihypertensives still form a sufficient component of the therapeutic treatments for high blood pressure that the clinician will encounter them routinely. However, the evidence of benefit, in terms of morbidity and mortality, is poorer for these groups than any other antihypertensive, and these classes of antihypertensives are of particular concern when it comes to the frailer patient who is at risk of falls. Multiple tools flag these medications as potentially inappropriate for frail older people, and a number of guidelines exist which can aid the clinician to review, and safely and appropriately de-prescribe.

**Fig. 1:**
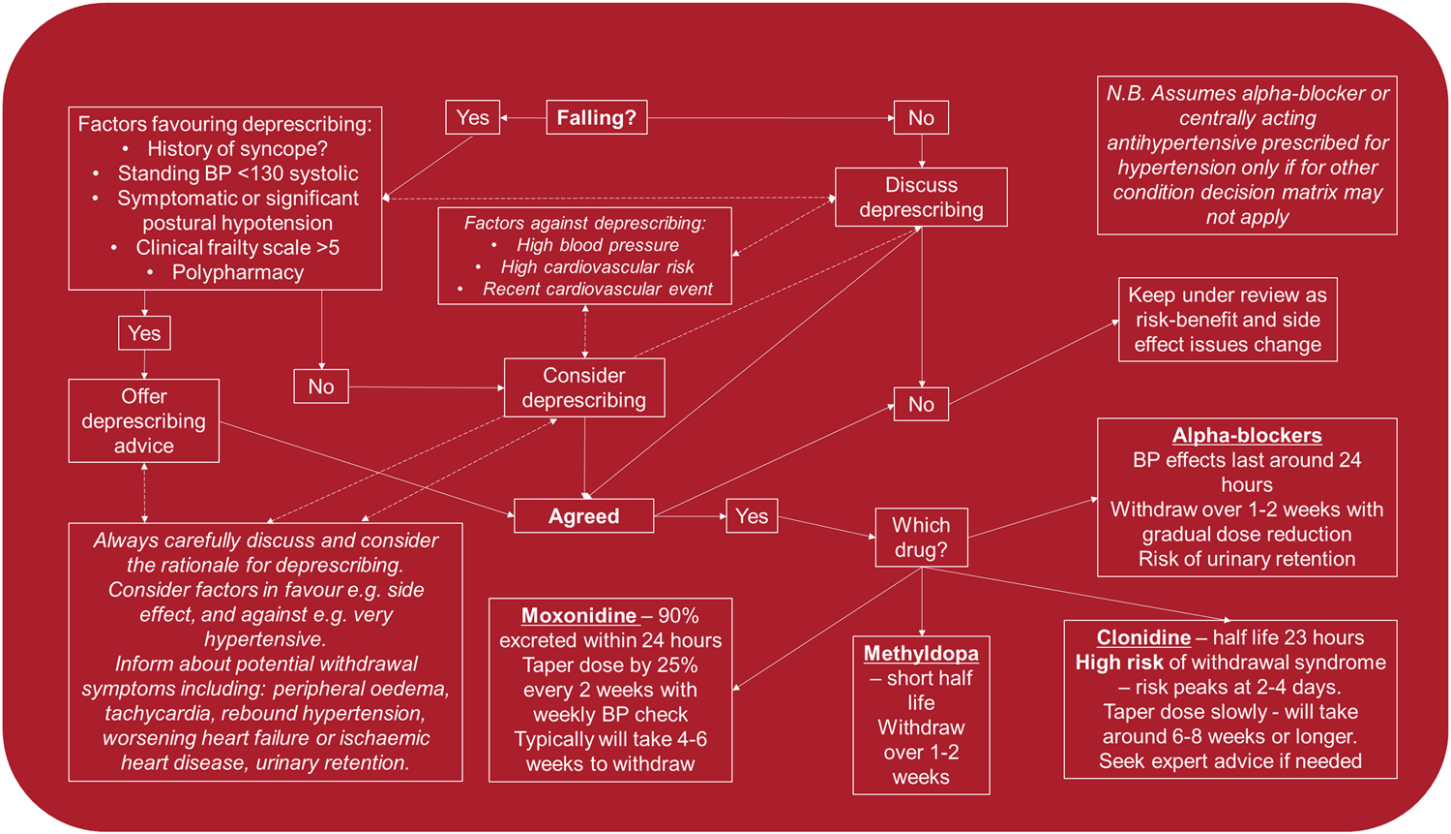
Alpha-blocker and centrally acting antihypertensive de-prescribing algorithm

In summary, some advice can be offered to the clinician managing a patient at risk of falls who is prescribed an alpha-blocker or centrally acting antihypertensive for high blood pressure:

- Centrally acting antihypertensives and alpha-blockers should be prioritised for de-prescribing in any older frailer individual at risk of falls unless there is an overwhelming indication for them to continue.
- Algorithm—We present a de-prescribing algorithm (Fig. 1).
- Shared decision-making—Individualise treatment and treatment goals, whenever possible, guided by patient priorities, symptoms, and individual risk profiles.
- Confirm the diagnosis—Look for orthostatic hypotension—measure postural blood pressures, then look again.
- Assess frailty and function—Consider more detailed assessment using an approach like comprehensive geriatric assessment.
- Consider contraindications—Are there any concerning symptoms or signs, e.g. syncope, bradyarrhythmias, heart failure? De-prescribe if a clear contraindication is present.
- Assess side effects—Consider non-cardiovascular side effects, such as sedation, depression, etc.
- Deprescribing*—*Slow reduction to avoid withdrawal syndrome. Monitor blood pressure.

## Future perspectives

Many clinicians are still hesitant when it comes to de-prescribing. Concerns about withdrawal syndromes and patient beliefs about the need to continue blood pressure medications indefinitely are often cited as issues. Future research should examine how clinicians and patients can be helped and supported to de-prescribe safely and appropriately.
